# The Thermal Structural Transition of Alpha-Crystallin Modulates Subunit Interactions and Increases Protein Solubility

**DOI:** 10.1371/journal.pone.0030705

**Published:** 2012-02-07

**Authors:** Giuseppe Maulucci, Marco De Spirito, Giuseppe Arcovito, Massimiliano Papi

**Affiliations:** Istituto di Fisica, Università Cattolica del Sacro Cuore, Roma, Italy; National Institute for Medical Research, Medical Research Council, United Kingdom

## Abstract

**Background:**

Alpha crystallin is an oligomer composed of two types of subunits, alpha-A and alpha-B crystallin, and is the major constituent of human lens. The temperature induced condensation of alpha-crystallin, the main cause for eye lens opacification (cataract), is a two step-process, a nucleation followed by an aggregation phase, and a protective effect towards the aggregation is exhibited over the alpha crystallin phase transition temperature (*Tc* = 318.16 K).

**Methods/Results:**

To investigate if a modulation of the subunit interactions over Tc could trigger the protective mechanism towards the aggregation, we followed, by using simultaneously static and dynamic light scattering, the temperature induced condensation of alpha-crystallin. By developing a mathematical model able to uncouple the nucleation and aggregation processes, we find a previously unobserved transition in the nucleation rate constant. Its temperature dependence allows to determine fundamental structural parameters, the chemical potential (Δμ) and the interfacial tension (γ) of the aggregating phase, that characterize subunit interactions.

**Conclusions/General Significance:**

The decrease of both Δμ and γ at *Tc*, and a relative increase in solubility, reveal a significative decrease in the strenght of alpha-crystallin subunits interactions, which protects from supramolecolar condensation in hypertermic conditions. On the whole, we suggest a general approach able to understand the structural and kinetic mechanisms involved in aggregation-related diseases and in drugs development and testing.

## Introduction

Cataract is the most common cause of blindness, and, therefore, of enormous medical and economical relevance worldwide. The social impact and economic cost of cataract have motivated extensive research on the lens and an enormous amount of knowledge has been accumulated [1]. Pathological studies of cataractous lenses have revealed that cataracts are composed of protein aggregates that precipitate in eye lens cells. The prevalent proteins within the eye lens are the crystallins. Lens transparency is thought to be maintained by a liquid-like, short range order present in highly concentrated solutions of these proteins [2],[3]. In mammals, there are three classes of crystallins denoted *α*, *β*, and *γ* of which *α*-crystallin is the most abundant. *α*-crystallin is an oligomer, having a molecular mass of about 800–1200 kDa, composed of two types of subunits, *α*A and *α*B crystallins, each having a molecular mass of about 20 kDa and *α*A crystallins in a ratio of 3 to 1 with respect to *α*B [4]. Differential scanning calorimetric studies on *α*-crystallin [5] show two endothermic transitions, a first ranging from 308 K to 324 K, peaked at *Tc* = 318.16 K and a second major transition peaked at T_IIC_ = 333.16 K. Near the biologically relevant transition at *Tc* [6] alpha-crystallin undergoes a minor change in its tertiary structure accompanying the exposure of its hydrophobic surfaces [7],[8].

The increase in light scattering in old and cataractous lenses can be ascribed to alterations in lens crystallins interactions due to age related post-translational modification of *α*-crystallin [9]–[12]. The alterations are triggered by lens cells exposition to elevated temperatures or other stress factors like Ca^2+^ ions, that disrupt the liquid-like molecular order and promote the formation of large scattering particles[13], [14] following pathways that include both changes in the secondary structure and in the state of assembly [15],[16]. Preliminary investigations on the temperature-induced alpha-crystallin aggregation showed the production of different heat-modified alpha-crystallin forms [17],[18]. At temperatures larger than *Tc* the kinetic pattern of the alpha-crystallin aggregation and the structural features of the clusters can be described according to the reaction limited cluster-cluster aggregation theory (RLCA) [18]. Growth kinetics occurs as a two step-process: a nucleation phase, in which basic aggregation units, the high molecular weight forms of alpha-crystallin (HMW) [19],[20] are initially formed, and an aggregation phase, in which HMWs diffuse, collide and form rather compact fractal aggregates (with a characteristic fractal dimension d_f_ = 2.15). Although the final morphology of the aggregates is similar [18] the aggregation kinetics are completely different below and above *Tc*, together with the size of the HMW, and their repulsive energy barrier (Eb). An abrupt increase in Eb above *Tc* reveals a mechanism that markedly protects from aggregation preserving the transparency of the lens [18]. However, the structural modification which occurs at *Tc*, and its relationship with the exhibited protective effect is still missing.

Here, we investigate if a modulation of the subunit interactions over Tc could trigger the protective mechanism towards the aggregation. To this aim we followed, by using static and dynamic light scattering, the temperature induced condensation of alpha-crystallin. Using a mathematical model which uncouples the nucleation phase and the aggregation phase, we find a previously unobserved transition in the nucleation rate constant. The analysis of nucleation rate constant, according to the classical nucleation theory, allows to rule out a structural modification which leads to a decrease in *α* -crystallin subunits interaction strengths, and a correspondent increase in alpha crystallin solubility which ultimately protects from supramolecolar condensation.

## Materials and Methods

### 1. Kinetic model of alpha crystallin aggregation

Population balances (PBE) are general equations describing the time evolution of CMD, applicable to a variety of particulate systems [21]. Aggregation in homogeneously mixed colloidal dispersions can conveniently be described by PBE, which use mass as the internal coordinate for representing aggregates undergoing birth and death events. These events lead to the formation and disappearance of aggregates of mass *m*. Indicating with *n_i_* (t) the number of aggregates of mass *m = i m_0_* at time *t* (i.e. the CMD) population balance equations have the following form:
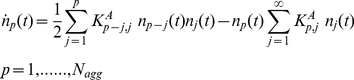
(1)where the two terms on the right-hand side represent the rate of birth and death of units of mass *m = i m_0_* per unit volume, respectively. The first one represents the production of aggregates of mass *m = i m_0_* by aggregation of two smaller aggregates of mass *m*′ and *m*−*m*′, while the second considers the loss of particles of mass *m* due to aggregation with any other aggregate of mass *m*′. *K_ij_* is the aggregation rate between two particles of mass *i m_0_* and *jm_0_*.

However, the application of equation (1) to proteins systems is often insufficient because it lacks the modellization of diverse protein related phenomena, like nucleation of basic aggregating units.

**Figure 1:**
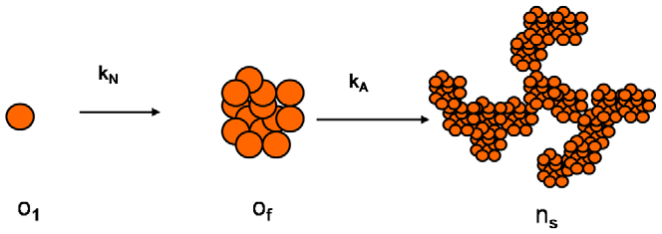
Schematic representation of the nucleation-aggregation process. The monomers *o_1_* form protein nuclei *o_f_* that act as a basic unit to direct the further growth of aggregates *n_s_*.

Here, according to our precedent findings [18], we model the formation of the basic aggregation units (HMW) as a nucleative mechanism, characterized by the initial formation of small, localized nuclei of proteins within the solvent, as a result of spontaneous density or composition fluctuations. When nuclei grow to a critical size, the aggregate starts to form spontaneously (Figure **1**). To include the nucleation mechanism in the PBEs (eq. (1)), we followed the Becker-Doring nucleation model from the field of atmospheric science [22]. Accordingly, the native alpha crystalline oligomers *o_1_*, having mass *m_0_*, react with one another as well as with different size nuclei so as to become larger clusters (Figure **1**). The reactions between larger nuclei are negligible because their early concentrations and diffusivities are relatively low and small, respectively, as compared with the monomers. As nuclei grow, their chemical potentials drop, yet the surface tension to form new phases rises. Hence, it exists a condition with minimum Gibbs free energy corresponding to the size of a critical nucleus *o_f_*, *M_C_*  = *N_c_m_0_* [23]. Any aggregates larger than the critical nucleus would convert into the basic unit of the aggregation. Therefore, indicating with *o_s_ (t)* the number of the growing nuclei of mass *m = s m_0_* at time *t* and indicating with *n_p_ (t)* the number of the aggregates of mass *m = p M_C_ = p N_C_ m_0_* at time *t*, we obtain the following modified form of the population balance equations (1):
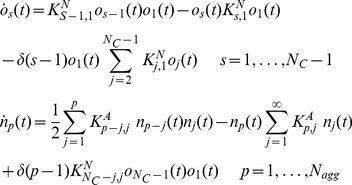
(2)where the two terms in the first equation on the right-hand side represent the rate of birth and death per unit volume of units of nuclei, of mass *m = s m_0_*, respectively, where 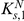 are the nucleation rates between native protein oligomers and nuclei of mass *m = s m_0_*. The two terms in the second equation on the right-hand side represent the rate of birth and death per unit volume of the aggregating clusters, of mass *m = p M_C_ = p N_C_ m_0_*, where 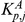 are the aggregation rates between clusters of mass *pm_0_* and *jm_0_* respectively. The third term represents all the oligomers larger than the critical nucleus that are converting into the basic unit of the aggregation. One of the first modellization of nucleation kinetics was applied in sickle-cell hemoglobin gelation [24]. The authors distinguished nucleation from polymerization. The nucleation process is described by a kinetic constant with a value less than one. The polymerization process, is instead described by a kinetic constant with a value more than one. That is, the kinetic process is assumed to be thermodynamically unfavorable until a critical nucleus is formed (nucleation), but then thermodynamically favorable during polymerization. In our case, there are not assumptions on the relationship between the nucleation and aggregation rate.

In the nucleation-aggregation model the averages 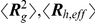 and *I (q)* of the cluster mass distribution become [25]–[27]:
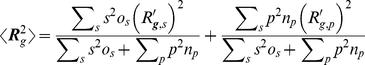
(3)Where 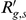 and 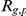 are the corresponding gyration radii of the oligomers of mass *s* and aggregates of mass *pm_0_*,
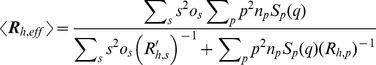
(4)Where *R′_h,s_*, *R_h,p_* are the corresponding hydrodynamic radii of the oligomers of mass *sm_0_* and aggregates of mass *pm_0_*, and *S_p_ (q)* represents the structure factor of the aggregates of mass *pm_0_*, and
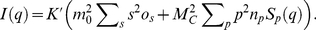
(5)
*R_h,p_*, the hydrodynamic radii of the oligomers of mass s and aggregates of mass *pm_0_* and fractal dimension *d_f_*, have the following expression [27]

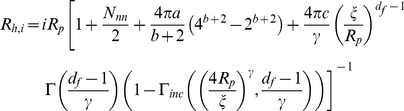
(6)Where *R_p_* is the hydrodynamic radius of the basic unit of the aggregation, 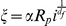 is the cut-off length, and the constant *α* equals 1.45 and 1.55 for DLCA and RLCA clusters, respectively. The parameters *a*, *b*, *c*, *N*_nn_, and *γ* are a function of the number of particles in the cluster and the empirical parameters *d*, *e*, and *f* take different values for the different parameters and Γ and Γ_inc_ are the Euler gamma function and incomplete gamma function, respectively. The values of the parameters are reported in [28].

The gyration radii 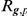 of the oligomers of mass *s* and aggregates of mass *p* have instead the following expression [29]

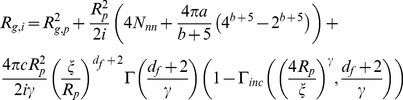
(7)where *R_g, p_* is the primary particle radius of gyration (for a sphere 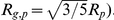


### 2.Preparation of *α*-crystallin suspensions

*α*-crystallin from bovine eye lens was prepared according to Andreasi et al. [19].

The *α*-crystallin fractions suspended in 10 mM Tris-HCl buffer, pH 7.4, were thoroughly mixed and pooled together. The purified protein was divided into aliquots and kept in the same buffer at 20°C until used. Just before the experiment, the samples were thawed and centrifuged at 5000 g (Eppendorf 5418) for 30 min at 4°C, and the supramolecular aggregates already formed were discarded. The super-natant was filtered through a 0:22_m Millipore low-retention filter directly into the measuring cuvette.

Protein concentration was determined by using an absorption coefficient of A _1 cm_^0.1%^ = 0,81 at 280 nm [2]. Aggregation of *α*-crystallin (1.0 mg/ml) was induced by quenching samples at the desiderated temperature and by the addition of 16 mM CaCl2. Indeed heating provokes the generation of particularly reactive isoforms of *α*-crystallin [5] and calcium ions stabilize the aggregates while they are forming and allow their continuous growth [13]. The whole set of measurements have been performed on different aliquots of the same sample. Five aggregations process for each temperature have been followed.

### 3. Static light scattering

Static light scattering [30] measures the time-averaged intensity *I(q)* scattered from a sample as a function of the scattering wave vector:
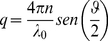
(8)Where *λ_0_* is the incident light wavelength, *n* is the refractive index of the solution, and *θ* is the scattering angle.

The measured scattering intensity from aggregating particles can be written as:
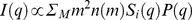
(9)where the contribution *M^2^S(qR_G_)* from a single cluster of mass *M* and radius of gyration *R_G_* is weighted over cluster-mass distribution *N(M)*, and *P(q)* is the form factor of the primary particle. The structure factor *S* of the aggregates can be obtained analytically by Fourier transforming the pair-correlation function of fractal objects [31]. Its normalized form with *S (0)* = 1 is given by the equation:
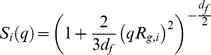
(10)where the dependence on the product *qR_G_* only follows the scale invariance of the cluster. Two asymptotic behaviours of the structure factor, corresponding to different experimental conditions, can be found during aggregation [26],[31],[32]:
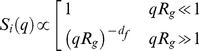
(11)When clusters can be considered like point sources, i.e., *qR_G_≪1*, static light-scattering intensity measurements can be used to determine the time evolution of the average cluster mass: 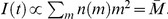 When most clusters are large enough to have *qR_G_≫1*, the fractal dimension *d_f_* can be directly determined by measuring scattered intensity versus wave vector 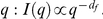 In the cross-over region *qR_G_∼1*, the full expression in eq.9 must be used.

### 4. Dynamic light scattering

Dynamic light scattering [33] measures the time autocorrelation function of the scattering intensity *I(t)*. The normalized autocorrelation function is defined as:
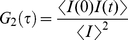
(12)where *τ* is the delay time and the angular brackets indicate the ensemble average.

The time dependence of the scattered intensity results from local density fluctuations as a consequence of the diffusive motion of the clusters. The autocorrelation function of these density fluctuations *g_1_(t)* can be derived from *G_2_* using the Siegert relation:
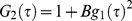
(13)where *B* is an instrumental constant.

For monodisperse point particles, the density autocorrelation function decays exponentially in time as 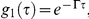 where the decay rate Γ depends on the particle translational diffusion coefficient according to Γ = *Dq^2^*. In the case of aggregating particles, deviations from the monoexponential decay are observed because of cluster polydispersity and rotational diffusion effects.

In this condition, the derivative of *g_1_* for 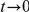 measures the average decay rate of the clusters:
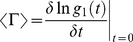
(14)To determine 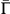 experimentally, we fitted the logarithm of the measured autocorrelation function *g*_1_, to a third-order polynomial, according to the cumulant expansion [34]:
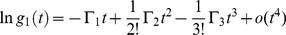
(15)where we assumed 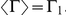


In aggregating systems, because of cluster-mass polydispersity, what we actually measure is an average effective diffusion coefficient that can be expressed as:
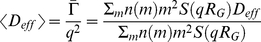
(16)The average effective hydrodynamic radius 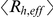 can be obtained using Einstein Relation
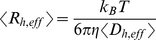
(17)


### 5. Light-scattering measurements

Static and dynamic light-scattering measurements were performed concurrently during *α*-crystallin aggregation by using a computer-interfaced scattering system ALV-5000 (ALV GmbH, Langen, Germany). A vertically polarized monochromatic light source at 632.8 nm produced by an NEC He-Ne 50 mW laser was used. The sample was contained in a cylindrical quartz cuvette (1-cm diameter) enclosed in a vat filled with toluene as optical matching fluid. Sample temperature was controlled within ±0.01°C by means of a Julabo HC Thermostat and measured with a Ptl00 thermometer. Photons scattered by the sample were revealed by a single photon photomultiplier mounted on the rotating arm of the goniometer.

The photopulses were sent to a 256-channel digital autocorrelator (ALV-5000) that performed a hardware autocorrelation function of the photopulses with a logarithmic spacing of delay times starting from 0.2 µs. Counts per second were used to measure the scattered intensity during the aggregation.

Data were collected from several scattering angles (usually eight) ranging from 30° to 150°, corresponding to wave vectors 0.46*10^5^<q<2.5*10^5^ cm^−1^. Because the measurements were performed during the aggregation process, data are a function of both scattering vector q and aggregation time *t*. The slow rate of the *α*-crystallin aggregation and the high values of scattered intensity usually allowed an average collecting time of 30 s, sufficient to obtain a good measure of the intensity autocorrelation function before the system could change significantly.

## Results and Discussion

### 1. Determination of nucleation and aggregation rate constant

**Figure 2:**
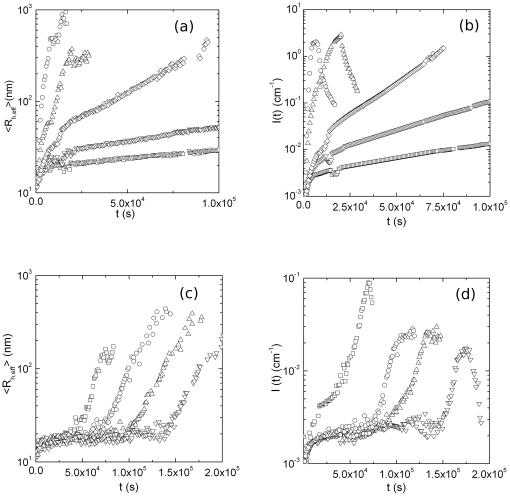
Temperature dependence of hydrodynamic radius and Rayleigh ratio of aggregating alpha crystallin suspensions. (a) Hydrodynamic radius and (b) Rayleigh Ratios versus aggregation time t of 1.0 mg/ml *α*-crystallin at 320.6 K (squares), 323.28 K (inverted triangles), 325.10 K (diamonds), 327.44 K (triangles) and 329.54 K (circles), above *Tc*. (c) Hydrodynamic radius and (d) Rayleigh Ratios measured at ***θ*** = 90° versus aggregation time t of 1.0 mg/ml *α*-crystallin at 310.63 K (inverted triangles), 311.60 K (triangles), 312.77 K (circles) and 314.45 K (squares), below *Tc*.

To characterize the extent of the aggregation process, we performed dynamic light scattering experiments by measuring the time evolution of the intensity weighted average hydrodynamic radius of the clusters 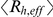 already reported in [18], determined according to Eq.(17), and of the Rayleigh Ratios *I (q)*, measured at *θ* = 90°, not reported in [18]. The results for samples at different temperatures above *Tc* = 318.16 K, are shown in Figure 2a–b. After an initial, fast, increase of 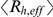, a second, slower, exponential growth, is observed. The first increase of 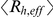 is ascribed to the initial conversion of the protein from the native to the heat- and calcium-induced conformers, that rapidly bind to form high molecular weight species (HMW) [18],[20]. The second exponential growth is instead consistent with an RLCA process where HMWs after a large number of collisions can stick together [19],[35]. By decreasing temperature below *Tc* the time evolution of the aggregation process undergoes to a dramatic modification (Figure 2c–d). Basic aggregation units are formed over a longer time and their average size is smaller, then an exponential increase of the hydrodynamic radius and the Rayleigh ratios, is still observed. Above and below *Tc* the aggregations are well characterized in the framework of RLCA theory: although the final morphology of the aggregates is similar, the aggregation kinetics seem completely different [18]. As we have seen we can compare the experimentally accessible quantities as *I(q)* and 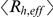 with those computed from the cluster mass distribution *n_i_* according to the nucleation-aggregation process. We developed an iterative procedure that, combining SLS and DLS data, namely *I(q)* and 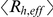, allows us to discriminate among different kinetic models. Indeed, by using kinetics evolution of both *I(q)* and 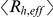 in the computation of PBE equations, we can account for two different averages of the distribution and we actually include information about the distribution width and shape. In order to compute *I(q)* and 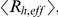 we need to know the structural features of the aggregates forming in the nucleation and in the aggregation process.

During the initial growth, ascribed to the nucleation process, we assume that particles have a spherical shape (*d_f, nuc_* = 3), and in the second step, corresponding to the aggregation process, clusters have a random fractal shape of dimension *d_f_* = 2.1 [18],[19]. Substituting eq.(10) in eq.(5) we will have as a theoretical expression for *I(q)*:
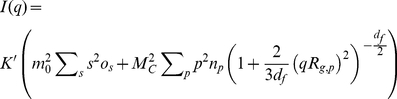
(18)with the *R_g, p_* given by relation (7) and *d_f_* = 2.1.

Further, substituting eq.(5) in eq.(4), we will have as a theoretical expression for 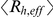:
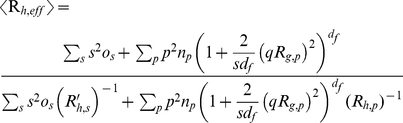
(19)with *d_f_* = 2.1, *R_g, p_* given by the relation (7), *R_h, p_*, and 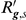 given by the relation (6) with respectively *d_f_* = 2.1 and *d_f_* = 3 . The nucleation constants between the nucleating i-mer and j-mer 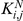


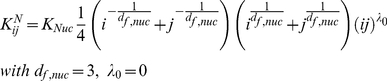
(20)Where *K_nuc_* represents the rate of formation of the first dimer in the nucleation process, *d_f,nuc_* is the fractal dimension of the clusters during the nucleation process, and *λ* is an exponent that accounts for variation in the aggregation efficiency of clusters due to their shape and therefore to their contact possibilities on their surface [36]. In our case, due to the small and almost spherical shape of the nucleation cluster, it is assumed equal to zero. Eq.20 is equal to *K_nuc_* for *i* = 1 and *j* = 1. It should be noted that it is also possible to recover all the other rate constants (i.e. monomer-dimer, dimer-tetramer) from eq.20. The aggregation constants between the aggregating cluster composed respectively of *i* and *j* nuclei, 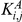 are
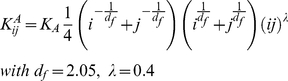
(21)Where *K_A_* represents the rate of dimerization of two critical nuclei, *d_f_* is the fractal dimension of the clusters during the aggregation process and *λ* = 0.4 according to [37]. All the other related rate constants can also be obtained from eq.21.

**Figure 3:**
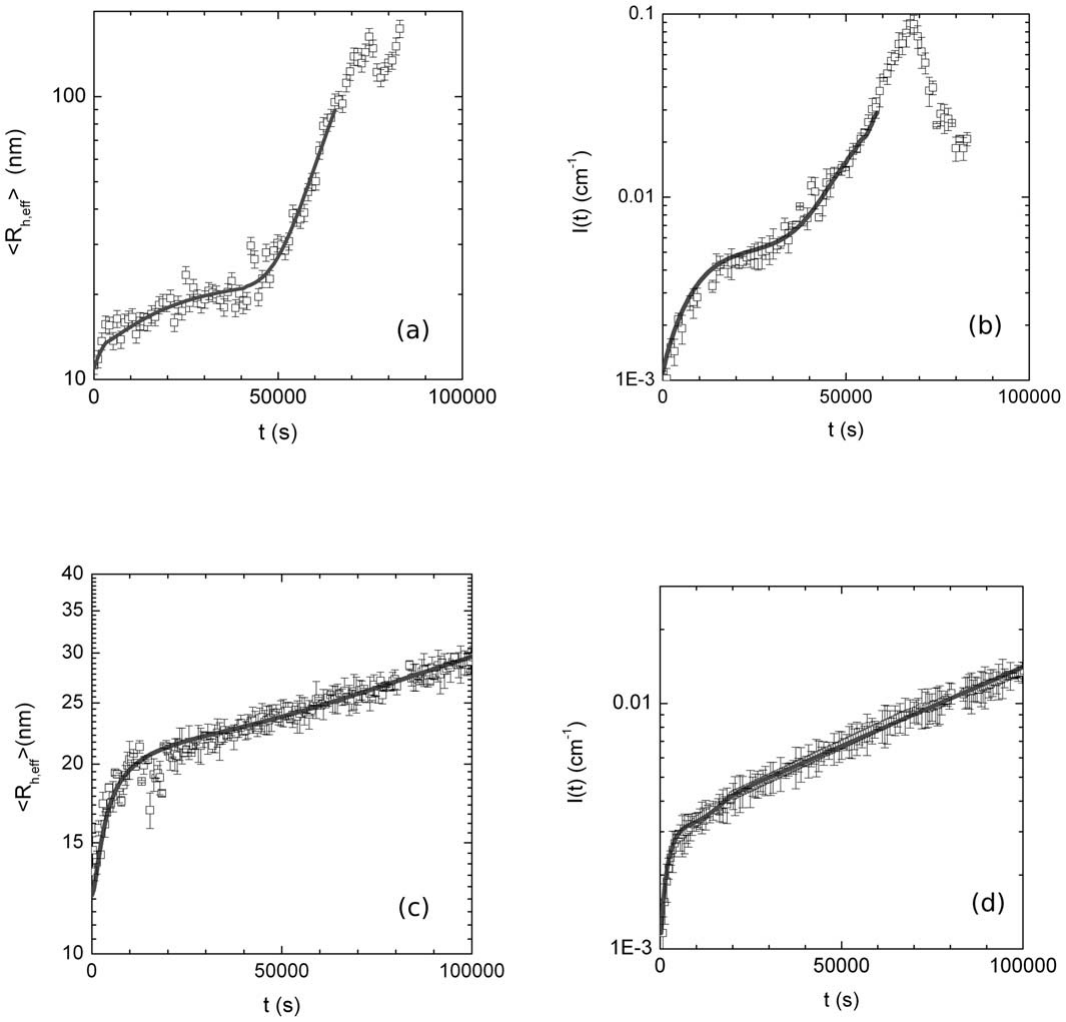
Iterative non-linear least squares fitting procedure of Hydrodynamic radius and Rayleigh Ratio by means of the nucleation- aggregation model. (a) Hydrodynamic radius and (b) Rayleigh Ratio measured at ***θ*** = 90° versus aggregation time t of *α*-crystallin at 314.45 K, below *Tc*. solid lines are the fit to the experimental data using the nucleation-aggregation model. (c) Hydrodynamic radius and (d) Rayleigh Ratio measured at ***θ*** = 90° versus aggregation time t of *α*-crystallin at 314.45 K, below *Tc*. solid lines are the fit to the experimental data using the nucleation-aggregation model.

We developed an iterative fitting procedure by means of non-linear least squares algorithms, to finally determine the parameters Nc, *K_nuc_* and *K_A_* The procedure consists in an iterative fit of I(q) (eq.18), which leads to a first estimation of the three unknown parameters. These are then used as starting guess for the fit of eq.19. If, after minimization, the parameters variation between the first and the second fitting procedure is less than 2%, the kinetic model is considered well in agreement with the experimental data. If not, the parameters *λ, λ_0_, d_f, nuc_, d_f_* are automatically varied, and the procedure restarts. In our case, for all the observed growth kinetics the values of these last parameters stabilize around the values reported in eq.20 and eq. 21. This suggests that no variations to the functional forms of 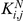 and 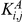 occur in these different environmental conditions.As an example, figure 3 shows the fit of eq. (18) to I(q) (Figure 3a) and the fit of eq. (19) to 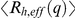 (figure 3b), measured at 314.45 K, below Tc. Figures 3c–d show the same fits to I(q) and 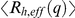 measured at 325.60 K, above Tc. It can be seen that equation 18 and 19 well recover experimental data, reinforcing further the model described as a nucleation phase followed by an aggregation phase [18],[19].

This procedure was repeated for all the kinetics followed while varying temperature. The results are reported in Table 1.

**Table 1:** *K_nuc_*, *K_A_* and *Rc* values obtained at different T. *Rc* was calculated from *Nc*.

T (K)	_Knuc(s_^−1^_M_^−1^_)_	_KA(s_^−1^_M_^−1^_)_	R_C_(nm)
310.6±0.01	(1.07±0.05)·10^−5^	(7.53±0.52)·10^−3^	22.2±1.1
311.6±0.01	(2.18±0.11)·10^−5^	(8.39±0.5)·10^−3^	23.5±1.4
312.7±0.01	(2.93±0.15)·10^−5^	(1.15±0.08)·10^−2^	20.8±1.3
314.4±0.01	(5.08±0.21)·10^−5^	(1.02±0.04)·10^−2^	22.2±1.4
320.6±0.01	(1.06±0.05)·10^−4^	(8.53±0.53)·10^−4^	26.0±1.7
323.8±0.01	(2.15±0.11)·10^−4^	(5.31±0.40)·10^−3^	25.9±1.7
325.3±0.01	(3.05±0.27)·10^−4^	(8.06±0.44)·10^−3^	29.1±1.7
327.3±0.01	(6.27±0.59)·10^−4^	(4.01±0.22)·10^−2^	29.1±1.5
329.5±0.01	(1.02±0.08)·10^−3^	(1.57±0.08)·10^−1^	29.1±1.9

### 2. Temperature dependence of nucleation and aggregation rates

**Figure 4:**
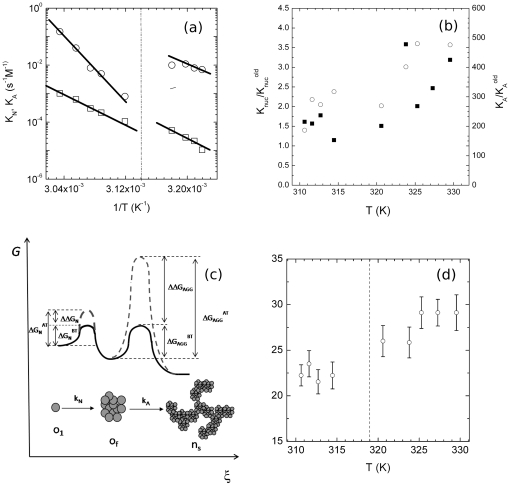
Temperature dependence of the rate constants, activation free energies and entropies of the nucleative and aggregative processes. (a) Arrhenius Plot of the aggregation and nucleation rates *K_nuc_* (squares), *K_A_* (circles) determined by the nucleation-aggregation model. Solid lines are exponential fit to the data. 1/*Tc* is indicated by a dashed line (b) Ratio of the nucleation rates determined in this article, *K_nuc_*, and 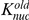 determined in [18] (open circles), togheter with the Ratio of the aggregation rates determined in this article, 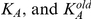 determined in [18] (squares). (c) Graphical representation of the nucleation-aggregation process in a free energy landscape below and above *Tc*. The free energy is reported as function of a reaction coordinate *ξ* that represents the progress of the process. At each minimum corresponds a stable state. Values 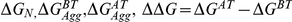 for are graphically shown. (d) Critical Radius *Rc* in function of temperature. *Tc* is indicated by a dashed line.

In Figure 4a we plotted the nucleation and aggregation rates, reported numerically in Table 1, by a semilogarithmic plot as a function of inverse temperature. Both rate constants exhibit an exponential trend till 1/T = 1/316 K^−1^: at this point they both abruptly break down, to then restart following the exponential trend with different slopes and prefactors. The jump between the two Arrhenius behaviours is in coincidence with the transition temperature of the quaternary structure of alpha-crystallin previously reported with different techniques [5],[6],[8],[18],[38].

With respect to the data reported in [18], in which only the transition in *K_A_* was revealed, our model shows a transition also for *K_nuc_*. Indeed the graphical estimation method used in [18] doesn't account correctly for the temporal overlapping between the nucleation and aggregation processes. To easily compare the rate constants determined by the two different estimation methods, we report the ratio between the nucleation rates *K_nuc_* and 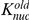 determined in [18], together with the ratio of the aggregation rates 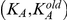 (figure 4b): the rate constants measured in [18] were systematically underestimated by a factor ∼2 for the nucleation constants and by a factor ∼300 for the aggregation constants. Indeed, the aggregation process starts when the first nuclei are formed, and if nucleation and aggregation constant have similar values, it is possible to estimate wrongly the naturally separated contributes. Such error, in our case, is higher above *Tc*, where nucleation and aggregation rates are effectively very similar (figure 4b).

Through the determination of rate constants at different temperatures, we can quantify the activation free energies and entropies, for both the nucleation and aggregation process. Indeed, temperature dependence of the rate constants is assumed to follow the Arrhenius law in the regions where the exponential trends are detected [39],
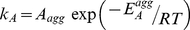
(24)

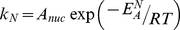
(25)Where *A_agg_*, *A_nuc_* are prefactors and 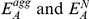 are respectively the energy of activation of formation of the first dimer in the nucleation process and the energy of activation of dimerization of the two critical nuclei.

In the two regions, the slopes of the straight line in Figure 4a determine the activation energies below and above *Tc*, which are reported in Table 2. 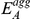 obtained in the previous article [18] were 33,8 and 137,6 kcal/mol, and are refined in this model, where temperature dependent underestimation of rate constant doesn't occur.

**Table 2:** Activation energies, activation entropies and activation free energies for the nucleation and aggregation process (values are expressed in kcal/M^−1^).

	Nucleation			Aggregation		
	E_A_	TΔS	ΔG	E_A_	TΔS	ΔG
**T<Tc**	62.8±1.8	50.6±3.0	12.2±1.1	42.4±1.2	34.8±2.0	7.6±0.7
**T>Tc**	60.7±1.8	47.4±2.8	13.3±1.2	131.0±3.9	115.2±6.9	15.8±1.4

### 3. Temperature dependence of the activation free energies and entropies of the nucleative and aggregative processes

We analyzed the results obtained for both the nucleative and aggregative dimerizations in the framework of the transition state theory [23],[40]. We assume that one monomer can bind to the other only when it is inside a reaction volume *v* with a characteristic size 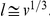 We also assume that the monomers entering the reaction volume can actually bind to the other only if these monomers are in the appropriate activation state. The probability of the occurrence of such a state is 
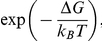
 where the change in free energy associated with the activation process is Δ*G* . Thus the dimerization rate can be written as
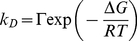
(26)Where Γ is the number of monomers entering the reaction volume per unit time. To estimate Γ, it must be noted note that the rate with which monomers enter a certain volume is equal to the rate with which they leave this same volume. The average number of monomers in a volume *v* at any moment of time is *cv*. These monomers are in a constant brownian motion and diffuse out of this volume in a time 
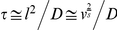
 to be replaced by others. Thus the number of monomers entering the reaction volume per unit time is *cν/τ* and therefore
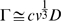
(27)It is reasonable to assume that the size of the reaction volume is of the order of the size of a monomer.

Substituting Eq. 26 into Eq. 27, and using the thermodynamic relation Δ*G = E_A_*−*T* Δ*S*, where *ΔS* is the change in the entropy associated with the activation process, we obtain
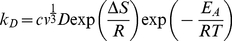
(28)Eq. 27 permits a physicochemical interpretation of the significance of the parameters *A* and *E_A_*, as obtained from the experimental measurements of *k_D_*(T). One can see indeed, by comparison of Eq. 27 and Eq. 23–24, that the activation entropy *ΔS* is related to the parameter *A* by the relation
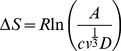
(29)In the case of the nucleation process the diffusion coefficient *D* (of the free alpha crystalline oligomer) is 3.6 10^−7^ cm^2^/sec^−1^ (for R_H_ = 10 nm). The reaction volume size 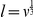 can be assumed to be of the order of the dimension of the *α*-crystallin, namely 10 nm. Thus, we estimated the changes in the entropy associated with the activation process below and above *Tc*, both for the nucleation and the aggregation, namely 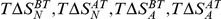 at 300 K, reported in Table 2. Although the values used to calculate the activation entropy are not known, especially in this last case, even a factor of 10 uncertainty in the magnitude of 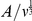 introduces an error of only 2.7RT = 1.6 kcal/mol in *T*Δ*S*, which is less than 4% of the total values. Note that, in comparison with the uncertainty in 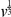, the error in the numerical value of *A* caused by a poorly known monomer concentration c produces an insignificant effect on the deduced value of the activation entropy Δ*S* [40]. In Table 2 changes in free energy associated with the activation processes are also reported, Δ*G = E_A_*−*T* Δ*S* calculated at 300 K. The free energies associated with the activation processes are ∼10 kcal/mol, which is a relatively small quantity, so that the probability for an activated state occurring, 
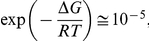
 is sufficiently large for the reaction to take place within the observed rate. However, this small free energy originates from the difference between the much larger activation energy and the entropy contribution, which goes from 40 to 130 Kcal/mol. The change in the free energy of activation with temperature, ΔΔ*G = *Δ*G^AT^−*Δ*G^BT^*, is 1.1 kcal/mol for the nucleation process and 8.2 Kcal/mol for the aggregation process. Below *Tc*, the free energy of activation of the nucleation process is nearly two times the free energy of activation of the aggregation process. Above *Tc*, while Δ*G_N_* stays almost unchanged, 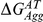, the free energy of activation of the aggregation process above *Tc*, is nearly two times larger than 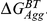 Therefore, the probability that an activated state occurs in the nucleation process is nearly the same above and below *Tc*, whereas the probability that an activated state occurs in the aggregation process switches from 
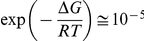
 down to 
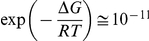
 at *Tc*. In Figure 4c, a schematic representation of the nucleation-aggregation process in a free energy landscape below and above *Tc* is reported. The free energy is reported as function of a reaction coordinate ξ that represents the progress of the process. At each minimum corresponds a stable state. Values for 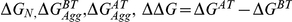 are graphically shown. It is clear that activated states above and below *Tc* differ significantly in both energy and entropy, suggesting a substantial difference in structure: indeed, as reported in Figure 4d, in correspondence of *Tc*, critical nuclei increase their radii from ∼23 nm to ∼28 nm. Above *Tc*, *Nc* is two times larger than below *Tc*, and we could estimate that the number of critical nuclei is near 1/2 of the number of critical nuclei below *Tc*, because the total mass is conserved and the nucleation rate does not vary appreciably. Thus, *Nc* increases, leading to the formation of a lesser number of critical nuclei.

### 4. Temperature dependence of protein-solution interfacial tension and the nucleus chemical potential

Thermodynamic structural features, as the protein-solution interfacial tension *γ* and the nucleus density times the nucleus chemical potential *ρ*Δ*μ*, above and below *Tc*, can be recovered from the knowledge of R* and ΔG^*^ following the classical nucleation theory. Classical nucleation theory (CNT) [39] expresses the rate per unit volume *k_N_* as the product of an exponential factor and a pre-exponential factor *A*

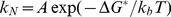
(30)The exponential factor is 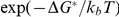 where ΔG^*^ is the free energy cost of creating the critical nucleus, the nucleus at the top of the barrier.

**Figure 5:**
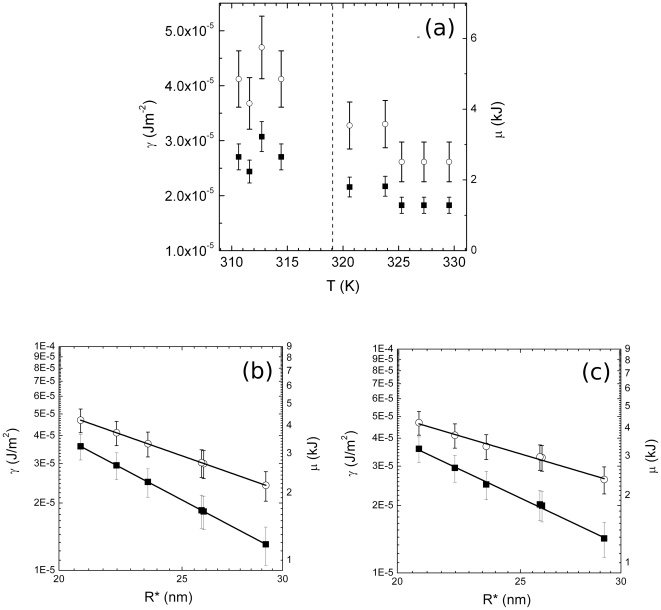
Temperature dependence of structural and thermodynamic properties of nuclei. (**a**) Temperature dependence of *γ*, the protein-solution interfacial tension (open circles) and Δ*μ* (full squares), the chemical potential difference between the protein phase and the solvent phase. *Tc* is indicated by a dashed line. Error bars are calculated according the propagation of uncertainty theorem. (b) Double logarithmic plot of the interfacial tension *γ* (open circles) and chemical potential Δ*μ* (squares) versus the critical radius of the new phase R*. Δ*μ* and *γ* were calculated assuming a constant value of the nucleation free energy of activation ΔG.^*^ Solid lines are power fit to the data. (c) Double logarithmic plot of the interfacial tension *γ* (open circles) and chemical potential Δ*μ* (squares) versus the critical radius of the new phase R*. Δ*μ* and *γ* were calculated accounting for the observed ΔG^*^ transition at *Tc*. Solid lines are power fit to the data.

CNT treats the nucleus as if it were a macroscopic phase. If we restrict ourselves to the nucleation of one fluid inside the bulk of another phase, then the nucleus is spherical and its free energy has just two terms: a bulk and a surface term. If the nucleus has a radius *R* then the bulk term is the free energy change involved in creating a sphere of radius *R* of the new phase. The surface term is the free-energy cost of the interface at the surface of this sphere. Thus the free energy is
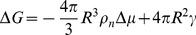
(31)Where Δ*μ* is the difference between the chemical potential of the phase where the nucleus is forming, and the chemical potential of the phase nucleating, *γ* is the interfacial tension, *ρ_n_*_is_ the number density of the nucleating phases. The free energy at the top of the barrier ΔG^*^ is easily found by setting the derivative of Δ*G* to zero. Then we have
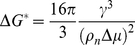
(32)This occurs for a critical nucleus of radius
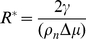
(33)The minimum (reversible) work of nucleus formation is maximized for R∼R*. The critical nucleus is therefore in unstable equilibrium, i.e. nuclei larger than R* grow spontaneously. Thus, in order for the new phase to be formed (i.e. irreversible aggregation to occur), the system must first overcome a free energy barrier and form a critical nucleus. Thereafter, the new phase occurs spontaneously (i.e. irreversible aggregation starts). Knowing ΔG^*^ and R* is therefore possible to determine *γ* and Δ*μ*. In our case we observe a decrease of both *γ* and Δ*μ* in correspondence of *Tc* (Figure 5a). *γ* ranges from values typical of mercury, below *Tc*, to values typical of ethanol. Thus, the free energy strength of the bonds that hold protein molecules together on the surface becomes ∼40% smaller above the transition temperature. Accordingly, being negligible the variation of *ρ* measured by SAXS data (Maulucci et al., in preparation), when the aggregation rate is reduced, the free energy strength of the bonds that hold protein molecules together in the bulk of the nucleus becomes nearly ∼50% smaller.

To rule out analytically the abrupt change of these thermodynamic quantities we proceed as follows: from equations (31) and (32) it is possible to see that if no transition occurs (i.e. ΔG^*^ is constant) 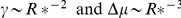 where *γ* is the interfacial tension and Δ*μ* is the chemical potential and R* the critical radius of the new phase. At the transition, the abrupt variation of the nucleation free energy of activation ΔG^*^ alter this trend leading to a variation of the characteristic exponent, i.e. 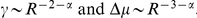


In fig. 5b a plot in double logarithmic scale of Δ*μ* (squares) and *γ* (open circles) vs R^*^(fig. 1B), calculated assuming a constant value of ΔG^*^ is reported. The exponents determined fitting *γ* values with a power law 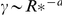 and Δ*μ* values with 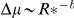 are, as expected, a = (1.99±0.05) and b = (3.00±0.06). In Fig. 5c the same plot of Δ*μ* and *γ* vs R*, calculated accounting for the observed ΔG^*^ transition at *Tc*, is reported. The exponents determined fitting *γ* values with a power law 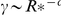 and Δ*μ* values with 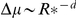 are now *c* = (1.68±0.05) and *d* = (2.68±0.06). Therefore *α* in both cases is equal to 0.32±0.06, and the exponent variation is a consequence of the phase transition.

### 5. Conclusions

The temperature induced condensation of alpha-crystallin, the main cause for lens opacification, is as a two step-process, a nucleation followed by an aggregation phase, and a protective effect towards the aggregation is exhibited over the alpha crystallin phase transition temperature (*Tc* = 318.16 K). In this work we showed how a modulation of the subunit interactions over *Tc* triggers the protective mechanism towards its self- aggregation. At the transition temperature, protein interactions become looser. Indeed a decrease of both interfacial tension and chemical potential (Figure 5) is observed, which corresponds to a net increase of solubility of alpha crystallin 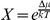 from ∼0.35 to ∼0.65. This increase leads critical nuclei to change their radii from ∼23 nm to ∼28 nm, determining a reduction of their number above *Tc*. The structural reconformation of the nucleus has a dramatic consequence in both the kinetics of nucleation and aggregation: the free energy barrier that must be overcome to form nuclei, and then aggregates, increases. Precipitation of the protein is therefore inhibited. Therefore an important missing link between the structural modification and the protective effect is found: the alpha crystallin phase transition, abruptly decreasing the strength of subunit interactions, markedly protects from aggregation above *Tc*, preserving the transparency of the lens.

On the whole, the simultaneous determination of kinetic and thermodynamic quantities, using population balance equations, give a mathematical framework useful to develop kinetic models of the aggregation processes, and can be a valuable tool in characterizing effects of several biomolecules on lens proteins supramolecular aggregation: biomolecules can inhibit the aggregation by reducing simply the kinetic constants, or by functionally altering the structure of HMW. Evaluating these different contributions may be decisive in understanding the mechanisms involved in aggregation-related diseases and in pharmaceutical development and testing.
